# Examining BPA’s Mechanisms of Action: The Role of c-Myc

**DOI:** 10.1289/ehp.123-A304

**Published:** 2015-12-01

**Authors:** Julia R. Barrett

**Affiliations:** Julia R. Barrett, MS, ELS, a Madison, WI–based science writer and editor, is a member of the National Association of Science Writers and the Board of Editors in the Life Sciences.

Bisphenol A (BPA) has shown an ability to disrupt endocrine signaling in some human and animal model studies, raising suspicions that it may be a factor in the development of endocrine-related disorders, including breast cancer.^1^ Animal studies appear to support that suspicion, but the precise molecular mechanisms by which BPA exposure may lead to cancer in humans are poorly understood.^2^^,^^3^ Previous studies have connected BPA exposure to molecular events in breast cells that are characteristic of cancer development.^4^ A new study reported in this issue of *EHP* implicates a gene further upstream of previously demonstrated mechanisms.^5^

BPA is used in a wide variety of products, including polycarbonate plastics, food and beverage containers, and coated papers (e.g., thermal receipts). Given the many sources of BPA, ongoing exposures are common, and most people carry low (nanomolar) concentrations of the compound in their bodies.^6^

**Figure:**
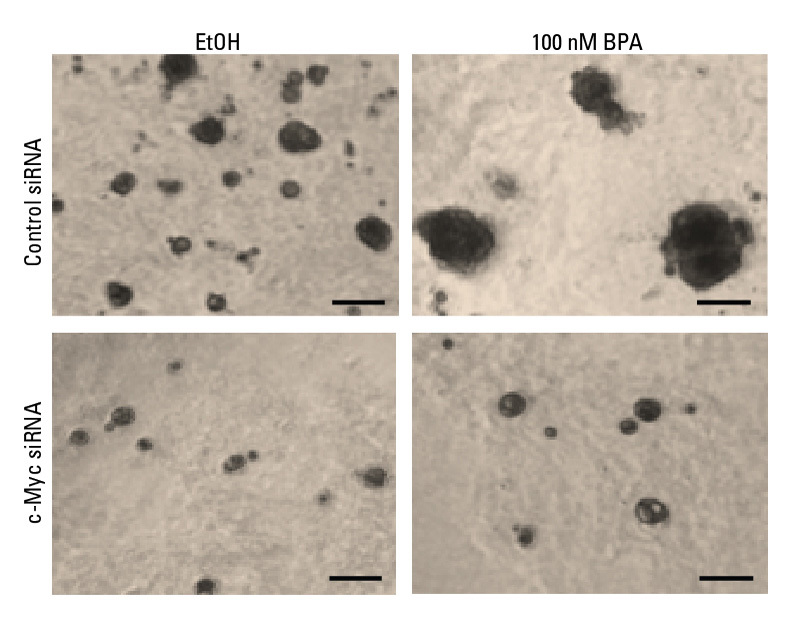
Compared with ethanol-treated control cells (top left), treatment with BPA (top right) accelerated cell proliferation, as shown by “spheroids” of cells in a 3D culture system. This effect was eliminated when c-Myc was silenced (bottom left and right). Source: Pfeifer et al. (2015)^5^

However, it’s difficult to determine the implications of those exposures for humans, because there is essentially no unexposed human population for comparison. BPA also has a short half-life in the human body, with most excreted within a day.^7^ Consequently, researchers have turned to *in vitro* cell culture systems to investigate cellular-level effects of BPA in a controlled manner.^4^

These investigations have led to numerous hypotheses about mechanisms by which BPA could harm breast tissue, but the conclusions are viewed with caution partly because experiments have often used much higher concentrations of BPA than have been measured in humans.^1^ Within the context of *in vitro* studies, BPA has been shown to disrupt cell signaling,^1^^,^^4^ which normally requires specific chemical messengers being at the right place at the right time to induce a wide array of downstream effects.^8^ These disruptions may alter the signals and the cellular processes they control, including those involved in cancer, such as the proliferation, mobility, growth, and programmed death of cells.^8^

Many of the endocrine-disrupting properties of BPA observed so far have been attributed to interaction with estrogen receptor alpha (ERα), and it is well known that BPA can induce proliferation of ERα-positive breast tumor cells.^1^ However, studies are lacking on the tumorigenic properties of BPA that may be unrelated to ER interactions. In the current study, the researchers therefore used three breast cancer cell lines that lacked ERα and a fourth that expressed it to investigate effects of BPA exposure at nanomolar concentrations.^5^

The researchers found that cells exposed to BPA had enhanced expression of the cancer-promoting protein c-Myc. In addition, BPA treatment was associated with higher levels of DNA-damaging reactive oxygen species, more DNA damage, and greater cell proliferation. Blocking transcription of the c-Myc gene (in other words, reducing production of the c-Myc protein) prevented these effects. The researchers concluded that c-Myc plays a key role in mediating BPA’s potential for inducing breast cancer at environmentally relevant levels.^5^

“This study is important for showing that BPA has very significant effects on breast health,” says study coauthor Mickey Hu, an associate professor of obstetrics and gynecology at Stanford Medical School. “Finding c-Myc as a target of BPA actually is a very important step for us to know how to prevent an adverse effect of BPA on human health.”

Hu cautions, however, that the findings should not be construed as saying that BPA will definitely cause breast cancer. “We cannot one hundred percent say that if you are exposed to BPA, you will develop breast cancer—that’s not true. There are a lot of genes involved in cancer development, and it’s a lot more complicated than what we saw [in this study],” he says.

Shanaz Dairkee, a senior scientist at the California Pacific Medical Center Research Institute, who studies the mechanistic effects of BPA and other estrogenic chemicals on healthy human breast cells *in vitro*,^4^^,^^9^ is intrigued by the effects induced within ERα-negative breast cells, since it suggests an impact on other tissues as well.

But Dairkee, who was not involved in the study, also notes that while numerous studies have found estrogenic chemicals to alter molecular, cellular, or tissue integrity, the jury is still out regarding their classification as carcinogens. Using experimental models to demonstrate an unequivocal role of environmental chemicals in human carcinogenesis “will require a data matrix incorporating a broader range of doses, population-based live test samples, and cancer end points than is currently out there,” she says.
